# Cellular uptake and antiproliferative effects of 11-oxo-eicosatetraenoic acid[S]

**DOI:** 10.1194/jlr.M040741

**Published:** 2013-11

**Authors:** Nathaniel W. Snyder, Sonia D. Revello, Xiaojing Liu, Suhong Zhang, Ian A. Blair

**Affiliations:** Centers for Cancer Pharmacology and Excellence in Environmental Toxicology, Department of Pharmacology, University of Pennsylvania, Philadelphia, PA

**Keywords:** cyclooxygenase, eicosanoids, cancer, exporters

## Abstract

Cyclooxygenases (COX) metabolize arachidonic acid (AA) to hydroxyeicosatetraenoic acids (HETE), which can then be oxidized by dehydrogenases, such as 15-hydroxyprostaglandin dehydrogenase (15-PGDH), to oxo-eicosatetraenoic acids (ETE). We have previously established that 11-oxo-eicosatetraenoic acid (oxo-ETE) and 15-oxo-ETE are COX-2/15-PGDH-derived metabolites. Stable isotope dilution (SID) chiral liquid chromatography coupled with electron capture atmospheric pressure chemical ionization (ECAPCI) single reaction monitoring (SRM) MS has been used to quantify uptake of 11-oxo-ETE and 15-oxo-ETE in both LoVo cells and human umbilical vein endothelial cells (HUVEC). Intracellular 11-oxo- and 15-oxo-ETE concentrations reached maximum levels within 1 h and declined rapidly, with significant quantitative differences in uptake between the LoVo cells and the HUVECs. Maximal intracellular concentrations of 11-oxo-ETE were 0.02 ng/4 × 10^5^ cells in the LoVo cells and 0.58 ng/4 × 10^5^ cells in the HUVECs. Conversely, maximal levels of 15-oxo-ETE were 0.21 ng/4 × 10^5^ in the LoVo cells and 0.01 ng/4 × 10^5^ in the HUVECs. The methyl esters of both 11-oxo- and 15-oxo-ETE increased the intracellular concentrations of the corresponding free oxo-ETEs by 3- to 8-fold. 11-oxo-ETE, 15-oxo-ETE, and their methyl esters inhibited proliferation in both HUVECs and LoVo cells at concentrations of 2–10 μM, with 11-oxo-ETE methyl ester being the most potent inhibitor. Cotreatment with probenecid, an inhibitor of multiple drug resistance transporters (MRP)1 and 4, increased the antiproliferative effect of 11-oxo-ETE methyl ester in LoVo cells and increased the intracellular concentration of 11-oxo-ETE from 0.05 ng/4 × 10^5^ cells to 0.18 ng/4 × 10^5^ cells. Therefore, this study has established that the COX-2/15-PGDH-derived eicosanoids 11-oxo- and 15-oxo-ETE enter target cells, that they inhibit cellular proliferation, and that their inhibitory effects are modulated by MRP exporters.

Arachidonic acid (AA) metabolism is implicated in cellular and physiologic regulation, inflammatory diseases, and cancer (1). In colon cancers, cyclooxygenase (COX)-2 expression is increased, and conversely, 15-prostaglandin dehydrogenase (PGDH) is downregulated (2). There is also evidence for COX-2/15-PGDH counterregulation in gastric, breast, and lung cancers (3–5). The magnitude of the upregulation/downregulation may even serve as an independent predictor of progression and survival (6, 7). This “proliferative switch” is hypothesized to increase tumorigenesis and angiogenesis via increased prostaglandin (PG) E_2_ formation (**Fig. 1A**) and a feed-forward loop for COX-2 (8, 9). However, COX-2-mediated AA metabolism also generates other eicosanoids, including 11- and 15-hydroperoxyeicosatetraenoic acids (HPETE) and, after reductive metabolism, the more stable 11- and 15-hydroxyeicosatetraenoic acids (HETE) (10, 11) (Fig. 1A, B). 15-HPETE is also a major product of the lipoxygenase pathway, through various 15- or 12/15-lipoxygenases (LOX; Fig. 1B) (12). 15-PGDH then oxidizes 11- or 15-HETE to the α,β-unsaturated ketone-containing oxo-eicosatetraenoic acids (ETE) (13, 14). Confirmation of the dehydrogenase pathway has been obtained using numerous experimental paradigms. 15-oxo-ETE was found as a major product of 15-PGDH-mediated oxidation of 15(*S*)-HETE in rabbit lung, as a major product of AA from mast cells, and as a major product of stenosed canine coronary arteries (15–17). In addition, either COX/15-PGDH-mediated or LOX/15-PGDH-mediated metabolism is involved in the formation of 15-oxo-ETE (Fig. 1B) (10, 14). Furthermore, 11-oxo- and 15-oxo-ETE have been detected in advanced human atherosclerotic lesions, although its route of formation was not examined in that study (18). Finally, we have demonstrated recently that 11-oxo-ETE is generated by COX/15-PGDH-mediated metabolism (13).

**Fig. 1.**
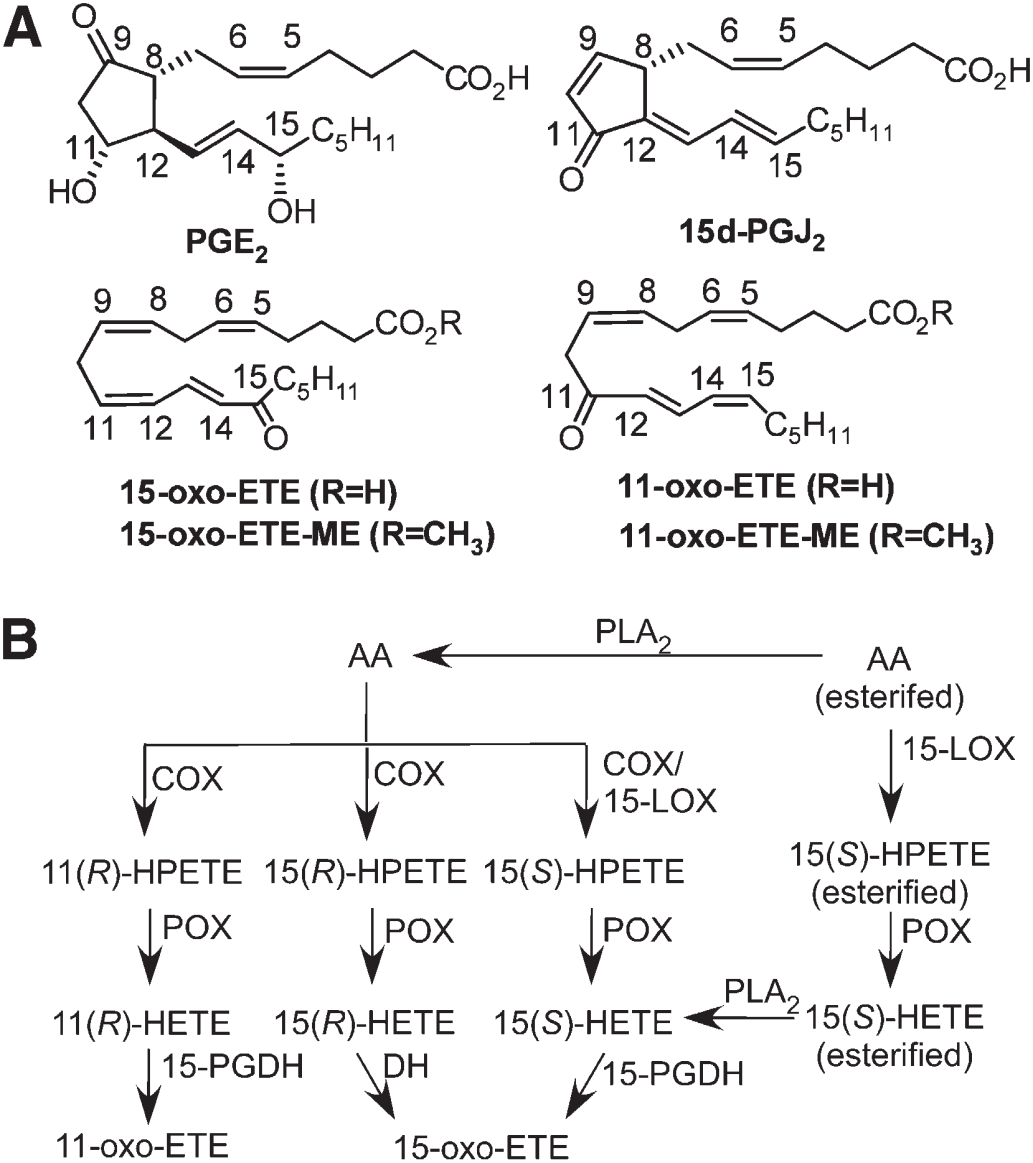
(A) Chemical structures of compounds used in this study, with PGE_2_ provided for comparison. (B) The currently elucidated COX-2/15-LOX- and 15-PGDH-dependent pathway for generation of 11-oxo-ETE and 15-oxo-ETE. 15-LOX, 15-lipoxygenase; cPLA_2_, cytosolic phospholipase A_2_; DH, dehydrogenase; PGH_2_, prostaglandin H_2_; POX, peroxidase.

In spite of significant evidence for the formation of 11- and 15-oxo-ETEs in vivo, the pharmacology of these endogenous metabolites has not been examined in detail. In contrast, the structurally related PG analog, 15-deoxy-Δ^12,14^-PGJ_2_ (15d-PGJ_2_; Fig. 1A) has been very extensively studied as an endogenous peroxisome proliferator-activated receptor-gamma (PPARγ) ligand, nuclear factor-kappa B (NF-κB) modulator, and redox signaling mediator (19–21). Antiproliferative and anti-inflammatory properties have also been examined for similar bioactive lipids, including 15-oxo-ETE (14), linoleic acid metabolites (22), a series of long-chain electrophilic fatty acids termed the EFOXs (23), and nitro-fatty acids (24). In the absence of a known G-protein coupled receptor, such as that activated by 5-oxo-ETE (25), these bioactive lipids are hypothesized to rely on intracellular targets. Therefore, understanding the availability of these compounds to the cytoplasmic space is of critical importance. This study was designed to test the cellular uptake, metabolism, and antiproliferative effects of 11-oxo-ETE in multiple cell models. We examined differential uptake of both 11-oxo- and 15-oxo-ETE between HUVECs and the LoVo colon cancer cell line. Using methyl-ester derivatives of 11-oxo-ETE and 15-oxo-ETE, we studied targeted intracellular delivery to HUVECs and LoVo cells. Finally, we investigated whether there was potentiation of the antiproliferative action of oxo-ETEs through targeted delivery or by pharmacological blockade of multidrug resistance protein (MRP) exporters.

## MATERIALS AND METHODS

### Chemicals and reagents

LC-MS Optima-grade hexanes, methanol, acetonitrile, isopropanol, protease inhibitor, and BCA protein quantification kit were obtained from Fisher Scientific (San Jose, CA). Dichloromethane, N,N-diisopropylethylamine (DIPEA), dimethyl sulfoxide (DMSO), and pentafluorobenzyl bromide (PFB) were from Sigma-Aldrich (St. Louis, MO). Probenecid was obtained from Enzo Life Sciences (Farmingdale, NY). Phosphate buffered saline (PBS) and 3-(N-morpholino) propanesulfonic acid (MOPS) were from Invitrogen (Carlsbad, CA). 11-oxo-ETE, 15-oxo-ETE, [^13^C_20_]15-oxo-ETE, as well as the methyl esters of 11-oxo-ETE (11-oxo-ETE-ME) and 15-oxo-ETE (15-oxo-ETE-ME) were prepared in house with standard procedures (13). Western Lightning ECL was obtained from Perkin Elmer (Waltham, MA). Fetal bovine serum (FBS) was obtained from Gemini Bioproducts (West Sacramento, CA). HUVECs, human arterial endothelial cells (HAEC), Medium 200, Low Serum Growth Supplement (LSGS), penicillin, streptomycin, F-12K media, and DMEM media were obtained from Invitrogen (Carlsbad, CA). LoVo, MCF-7, A549, and HCA-7 cell lines were obtained from American Type Culture Collection (ATCC) (Manassas, VA).

### Cell culture

LoVo and adenocarcinoma human alveolar epithelial (A549) cells were maintained in F-12K media supplemented with 2% FBS and 100,000 units/l penicillin and 100 mg/l streptomycin. HUVECs and HAECs were maintained in Medium 200 (Invitrogen) supplemented with the LSGS Kit on Collagen I-coated tissue culture dishes (Becton Dickinson, Bedford, MA). Human colonic adenocarcinoma (HCA-7) cells and the MCF-7 breast cancer cells were maintained in DMEM supplemented with 2% FBS and 100,000 units/l penicillin and 100 mg/l streptomycin. The relevant maintenance media was used for treatment unless otherwise indicated.

### Quantification of cellular uptake

LoVo cells and HUVECs were plated at 4 × 10^5^ cells/well in a 6-well tissue culture plate (Corning, Corning, NY) or in a 6-well collagen-coated 6-well plate (Becton Dickinson, Bedford, MA) and allowed to attach for 12 h. 11-oxo-ETE, 15-oxo-ETE, or the respective methyl-ester stocks were resuspended in media containing 0.25% DMSO at indicated concentrations. Either LoVo cells or HUVECs were treated for indicated time points with indicated compounds. Media was pipetted off; a 3 ml aliquot was spiked with 1 ng [^13^C_20_]15-oxo-ETE internal standard, and taken for analysis. The cells were rinsed four times with cold PBS, gently scraped into 3 ml of cold PBS, and spiked with 1 ng [^13^C_20_]15-oxo-ETE internal standard. The final rinse of cold PBS was taken and sampled as a control for residual 11-oxo- or 15-oxo-ETE. Extraction and derivatization has been described in detail elsewhere (26). Briefly, media was extracted with diethyl ether with 0.5% acetic acid with vigorous shaking, and the organic phase was separated and evaporated under nitrogen gas. Cells were extracted with dichloromethane/methanol (2:1 v/v with 0.5% acetic acid) with vigorous shaking and one freeze-thaw cycle; the organic phase was then removed and evaporated under nitrogen gas. After evaporation of the organic phases to dryness, samples were suspended in 100 µl DIPEA in acetonitrile (1:9 v/v) and 200 µl of PFB bromide in acetonitrile (1:19 v/v) and kept at room temperature for 30 min. The derivatized samples were dried down again under nitrogen, and then resuspended in 100 µl of hexane/ethanol (95:5 v/v) for stable isotope dilution (SID) chiral LC electron capture atmospheric pressure chemical ionization (ECAPCI) single reaction monitoring (SRM) MS analysis.

### Treatment of cells with [^13^C_20_]15-oxo-ETE

LoVo cells (5 × 10^6^) were cultured as described above and then treated with 10 mM [^13^C_20_]15-oxo-ETE. Cell and media fractions were pooled, and derivatization and analysis were performed as described above, except the internal standard was omitted.

### Liquid chromatography

A Water's Alliance 2690 HPLC system (Waters Ltd, Watford, Hertfordshire, UK) was used for liquid chromatography separations. The PFB derivatives of 11-oxo-ETE and 15-oxo-ETE were separated using a normal phase Chiralpak AD-H column (250 × 4.6 mm, 5µm; Daicel Chiral Technologies, Westchester, PA) with a 1 ml/min flow rate. Solvent A was hexanes and solvent B was isopropanol/hexane (6:4 v/v). Gradient composition was 2% B at 0 min, 2% B at 14.5 min, 12% B at 15 min, 90% B at 17 min, 90% B at 22 min, 2% B at 22.5 min, and 2% B at 29 min.

### Mass spectrometry

A Thermo Triple Stage Quadrupole (TSQ Quantum) mass spectrometer (Thermo Scientific) with an APCI source was operated in negative ion mode. The following transitions corresponding to each compound were monitored: 11-oxo-ETE-PFB, *m/z* 317→165 [collision energy (CE), 25 eV]; 15-oxo-ETE-PFB, *m/z* 317→113 (CE, 18 eV); [^13^C_20_]15-oxo-ETE-PFB, *m/z* 337→120 (CE, 18 eV). For absolute quantification of 15-oxo-ETE and 11-oxo-ETE, standard curves ranging from 0 to 2 ng and 0 to 4 ng, respectively, were generated in the same matrix under identical extraction conditions with pure compounds. Data analysis was performed using Xcalibur software (Thermo Scientific).

### BrdU incorporation assays

HUVECs, LoVo, HCA-7, and A549 cells were plated at 2,000 cells/well and allowed to attach for 12 h. Treatment media was prepared at indicated concentrations by serial dilution from the most concentrated stock, keeping constant 0.25% DMSO. Cells were treated for 24 h, and then spiked with BrdU for 6 h to allow incorporation into newly synthesized DNA. The assay was developed using a BrdU cell proliferation kit (Roche Diagnostics) according to the manufacturer's directions, and a UV-Vis plate reader (Bio-Rad, Hercules, CA).

### Western blots

Cells were collected from preconfluent cultures and lysed in RIPA buffer containing 1X protease inhibitor cocktail. Protein was quantified with a BCA kit. Thirty micrograms (30 μg) of protein lysate in reducing conditions was loaded into 4–12% gradient gel and run in MOPS buffer for 50 min at 200V. Proteins were transferred onto a nitrocellulose membrane overnight on ice at 30V. After blocking with 5% BSA in TBS/T, primary antibody was incubated overnight in blocking buffer. Primary antibodies for MRP1, MRP4, and GAPDH were, respectively, ab32574-100, ab56675, and ab8245 (Abcam). Secondary antibody was HRP-conjugated sheep anti-mouse from GE Life Sciences (NA9310). All antibodies were diluted in blocking buffer at 1:1,000. Visualization was accomplished with Western Lightning ECL in a digital developer (GE Healthcare).

### MTT proliferation assays

LoVo cells were plated at 2,000 cells/well and allowed to attach for 12 h. Treatment media was prepared at indicated concentrations by serial dilution from the most concentrated stock, keeping constant 0.25% DMSO. Probenecid was added from a concentrated stock to 1 mM treatment concentration. After indicated time points, media was replaced with fresh base media containing no FBS or pen/strep, and MTT was added to a final concentration of 2 mg/ml and allowed to incubate for 4 h. After incubation, all of the media was removed, and the MTT was eluted using pure isopropanol. The resulting absorbance was read at 565 nm in a 96-well plate using a UV-Vis plate reader (Bio-Rad).

### Statistical analysis

All statistical analyses were carried out using the GraphPad Prism 5 software package.

## RESULTS

### Intracellular 11-oxo-ETE was reduced in LoVo colon cancer cells versus human umbilical vein endothelial cells

To study the uptake and metabolism of 11-oxo-ETE, LoVo cells or HUVECs were incubated with 10 μM of 11-oxo-ETE, 10 μM of 15-oxo-ETE, or media with 0.25% DMSO vehicle for 4 h. Media and cells were collected at various time points. Quantification of the free 11-oxo- and 15-oxo-ETE was performed by stable isotope dilution chiral LC-SRM/ECAPI/MS with [^13^C_20_]15-oxo-ETE as the internal standard. Cells were carefully normalized to cell count used in the experiments and only allowed a minimum of time to attach in order to avoid excess growth. Cell volume determination would require lifting of the cells that, especially in the case of the collagen-attached, elongated HUVECs, could result in a cell volume change.

**Fig. 2.**
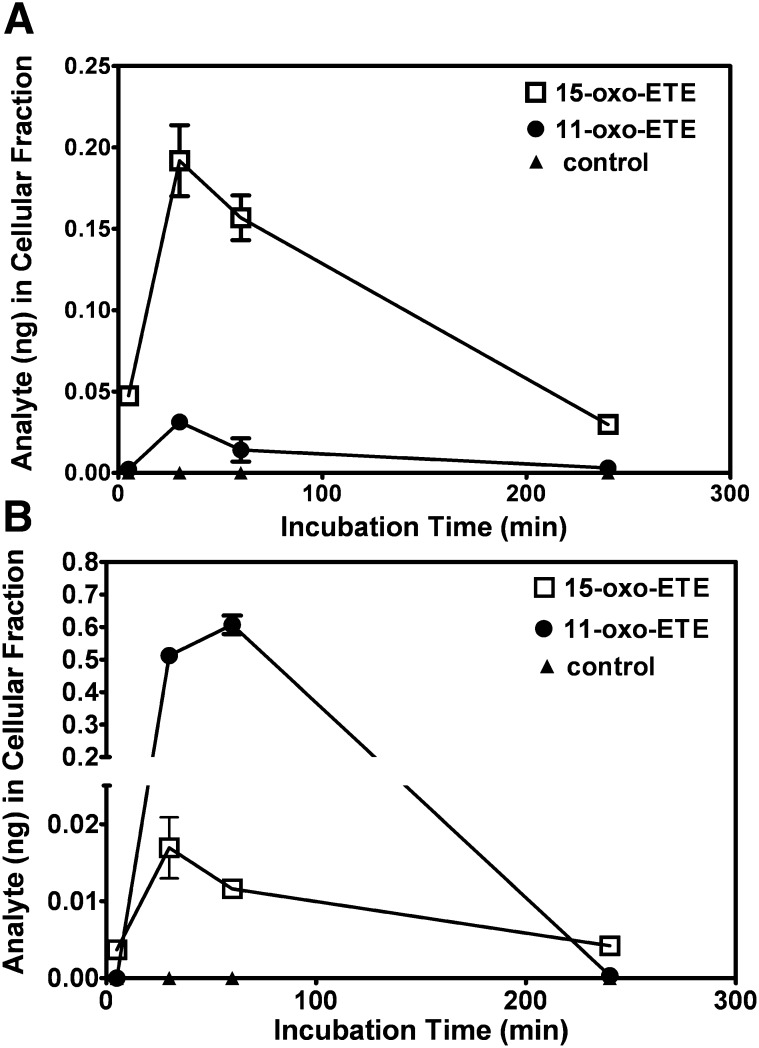
SID-LC-ECAPCI/SRM/MS quantification of uptake and distribution of 15-oxo-ETE and 11-oxo-ETE by (A) LoVo cells and (B) HUVECs over time. Cells were incubated at 37°C with 15-oxo-ETE (2 µM) or 11-oxo-ETE (2 µM) for 5, 30, 60, and 240 min. For each time point, cellular fractions were collected, extracted, spiked with [^13^C_20_]15-oxo-ETE, and then derivatized with PFB. Each time point is plotted as the mean of triplicates with SEM.

Intracellular concentrations of 11-oxo-ETE were reduced in the LoVo cells (**Fig. 2A**) compared with the HUVECs (Fig. 2B) at all time points examined. 15-oxo-ETE demonstrated the opposite trend, with greater intracellular amounts in LoVo cells (Fig. 2A) versus HUVECs (Fig. 2B). Maximal uptake of 11-oxo-ETE was achieved for LoVo cells and HUVECs at 30 min and 60 min, respectively. 15-oxo-ETE maximal uptake occurred at 30 min for both cell types. Clearance of the free 11-oxo-ETE occurred completely in both cell lines by 4 h, whereas 15-oxo-ETE was still detectable at that time. Treatment of the LoVo cells with [^13^C_20_]15-oxo-ETE did not cause the generation of endogenous 11-oxo-ETE or 15-oxo ETE as judged by comparison of the LC-MS chromatogram that was obtained from the cell suspension (supplementary Fig. III) with that obtained from the internal standard alone (supplementary Fig. I). This finding conclusively demonstrated that 11-oxo-ETE uptake and metabolism were significantly different between the LoVo cells (Fig. 2A) and HUVECs (Fig. 2B).

### 11-oxo-ETE inhibited BrdU incorporation across multiple cell lines with varying potency

**Fig. 3.**
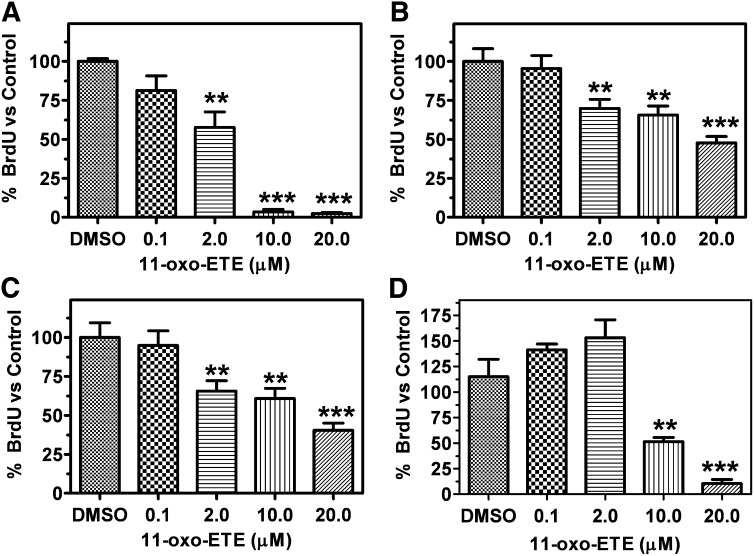
BrdU incorporation measured by ELISA in (A) HUVECs, (B) LoVo cells, (C) HCA-7 cells, and (D) A549 cells following 24 h treatment with indicated dose of 11-oxo-ETE in 0.25% DMSO compared with vehicle control. One-way ANOVA with post-hoc Dunnett's multiple comparison test versus vehicle control was used to assess statistical significance (*P < 0.05, **P < 0.01, ***P < 0.001). Data are plotted as the means (n = 3–8) with SEM bars, representative of at least two independent experiments.

BrdU incorporation assays were used to measure the antiproliferative effects of treatments with increasing doses of 11-oxo-ETE. The value obtained for vehicle treatment 0.25% DMSO was arbitrarily set at 100%. Multiple cancer cell lines were used, including LoVo, HCA-7, and A549 from colon, colon, and lung cancers, respectively. The same assay was conducted with HUVECs to allow comparison to our earlier work on 11-oxo- and 15-oxo-ETE (13, 14). Increasing doses showed a dose-dependent reduction in proliferation. HUVECs were the most sensitive to treatment (**Fig. 3A**) followed by the colon cancer lines LoVo (Fig. 3B) and HCA-7 (Fig. 3C). A549 lung cancer cells showed no significant response to treatment until higher doses of 11-oxo-ETE were used (Fig. 3D).

### 11-oxo-ETE-ME and 15-oxo-ETE-ME preferentially targeted the intracellular space

**Fig. 4.**
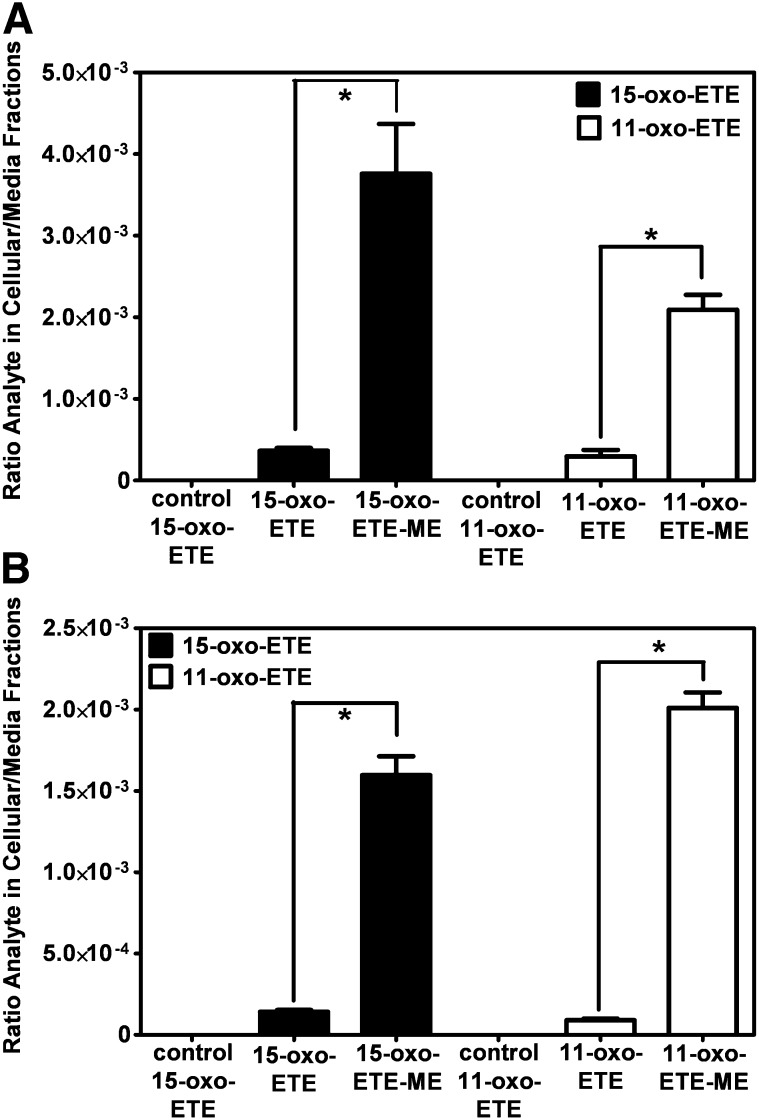
SID-LC-ECAPCI/SRM/MS quantification of the ratio of cellular/media 11-oxo- and 15-oxo-ETE in (A) LoVo cells and (B) HUVECs. Cells were incubated with 15-oxo-ETE (10 µM), 15-oxo-ME (10 µM), 11-oxo-ETE (10 µM), 11-oxo-ME (10 µM), or 0.25% DMSO vehicle control for 60 min. Cellular and media fractions were collected, extracted, spiked with [^13^C_20_]15-oxo-ETE, and then derivatized with PFB. Data are plotted as the means of triplicates with SEM of the ratio of analytes in the cellular over media fractions. Statistical significance was assessed by one-way ANOVA with post-hoc Bonferroni multiple comparison test (*P < 0.05).

To test the targeting of oxo-ETEs to the intracellular environment, 11-oxo-ETE-ME, 15-oxo-ETE-ME, 11-oxo-ETE, 15-oxo-ETE, and a vehicle control were incubated with LoVo cells and HUVECs for 60 min. Media and cells were then extracted, and free oxo-ETEs were quantified by LC-MS. The methyl esters significantly increased the levels of free oxo-ETEs in the cell over the amount in the media in both LoVo cells (**Fig. 4A**) and HUVECs (Fig. 4B). The free 15-oxo-ETE reached a higher intracellular concentration in the LoVo cells (Fig. 4A). In contrast, the 11-oxo-ETE was higher in the HUVECs (Fig. 4B). Essentially no 11-oxo-ETE or 15-oxo-ETE was detected in the LC-MS chromatogram from the combined fourth washes of the LoVo cells treated with 11-oxo-ETE, 15-oxo-ETE, 11-oxo-ETE-ME, or 15-oxo-ETE-ME (supplementary Fig. III) compared with a control internal standard alone (supplementary Fig. II). Similar results were obtained from HUVECs (data not shown). Furthermore, there was no detectable 11-oxo-ETE-ME or 15-oxo-ETE-ME in the fourth washes of the cells (data not shown). This finding confirmed that none of the 11-oxo-ETE or 15-oxo-ETE could have arisen from material left on the cell surface and suggested that the methyl-ester derivatives could provide a useful delivery strategy to target the intracellular environment for both 11-oxo- and 15-oxo-ETE.

### MTT assays over multiple days demonstrated antiproliferative effects for 11-oxo-ETE and 11-oxo-ETE-ME

**Fig. 5.**
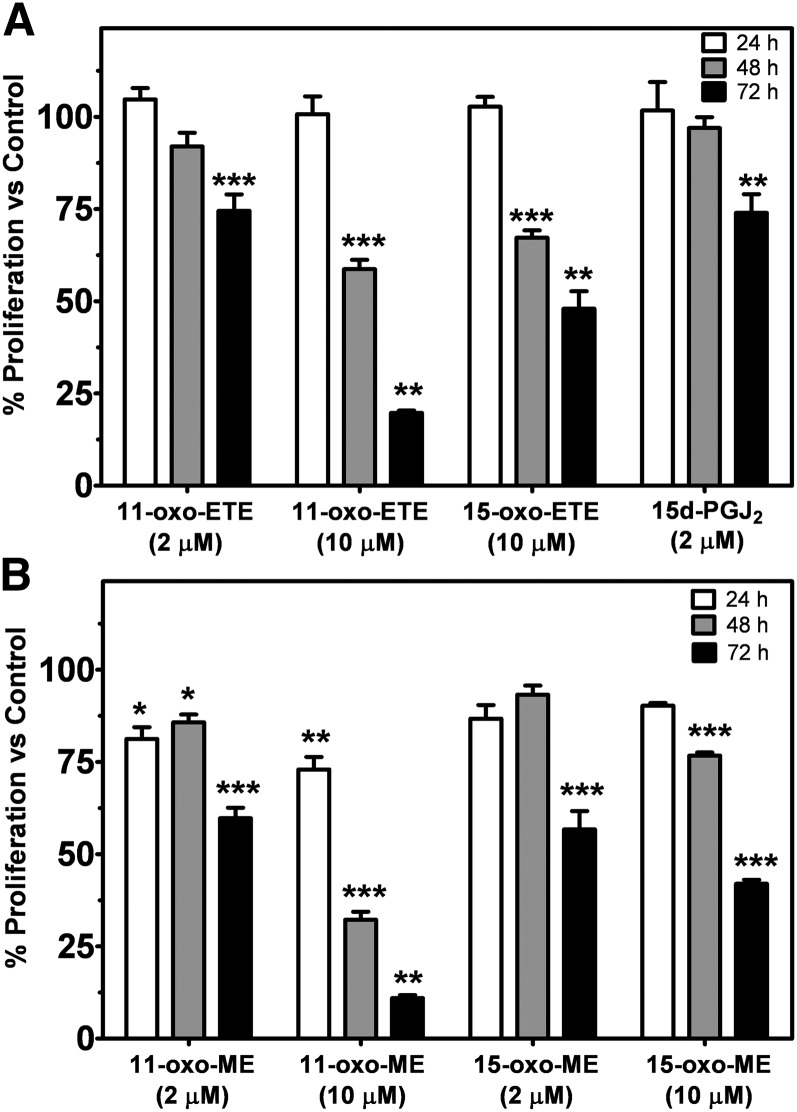
Cell proliferation measured by MTT assay with LoVo cells over multiple days with the indicated compounds in 0.25% DMSO compared with vehicle control. Treatments with the free acids are shown in (A), the methyl esters are shown in (B). Data are plotted as the means (n = 4) with SEM, representative of at least two independent experiments. Statistical significance was assessed by one-way ANOVA with post-hoc Bonferroni multiple comparison test against the respective vehicle control (*P < 0.05, **P < 0.01, ***P < 0.001).

To observe the antiproliferative effects of 11-oxo-ETE, 15-oxo-ETE, and their methyl esters, MTT assays were carried out over 72 h. Every 24 h, samples were collected, and the media was refreshed. Values obtained for vehicle treatment 0.25% DMSO was arbitrarily set at 100%. 15d-PGJ_2_ was included as a reference compound. 11-oxo-ETE dose dependently inhibited growth over multiple days (**Fig. 5A**). Furthermore, 11-oxo-ETE-ME reached significance for inhibition before the free 11-oxo-ETE (Fig. 5A). In all cases, by 72 h, significant antiproliferative effects were observed versus the vehicle control (Fig. 5A, B). Interestingly, 11-oxo-ETE-ME (Fig. 5B) was more potent than 11-oxo-ETE (Fig. 5A), causing a significant antiproliferative effect at all three time points.

### Transporter proteins MRP1/MRP4 were expressed in the more resistant cell lines

**Fig. 6.**
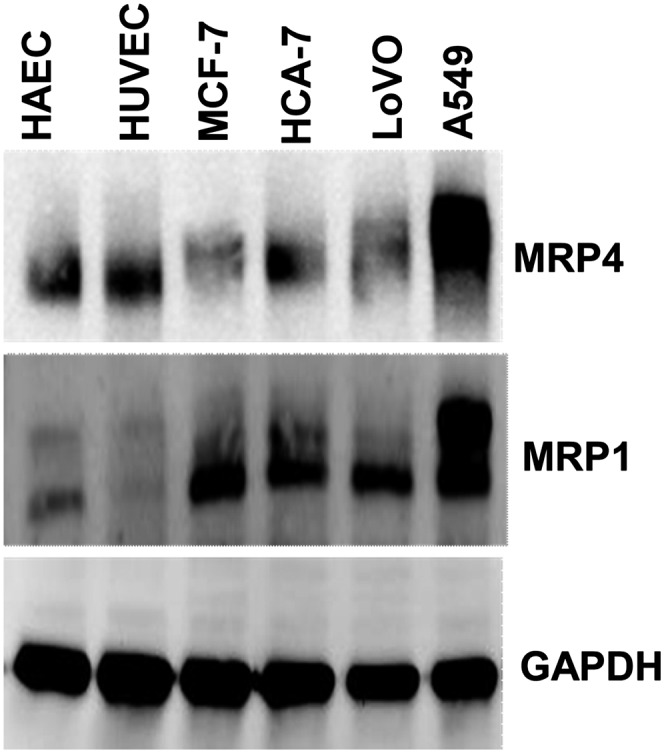
Western blots of HAEC, HUVEC, MCF-7 cells, HCA-7 cells, LoVo cells, and A549 cells for transporters MRP4 and MRP1, together with the loading control GAPDH. Increased expression of MRP1 was observed in the cancer cells lines (MCF-7, HCA-7, LoVo, A549) versus the endothelial cells (HAEC, HUVEC). MRP4 expression was detectable in all cell lines, with robust expression in A549 cells.

To help understand why there were differences in the intracellular 11-oxo-ETE concentrations, the expression of MRP1 and MRP4 membrane transporters was examined. MRP4 expression was robust in the A549 lung cells and significant in the two endothelial lines tested (HUVEC and HAEC), whereas expression of MRP1 was robust in all cancer lines (LoVo, HCA-7, MCF-7, and A549) compared with the two endothelial lines (**Fig. 6**). This finding suggested that increased MRP1 expression could have been a major determinant of the reduced cellular 11-oxo-ETE levels in LoVo cells (Fig. 2A) compared with the HUVECs (Fig. 2B). MRP1 has previously been implicated in PG export, especially in the context of cancer cell-dependent upregulation of tumor microenvironment PGE_2_ (27).

### Antiproliferative effects of 11-oxo-ETE-ME were increased with cotreatment of the drug transport inhibitor probenecid

**Fig. 7.**
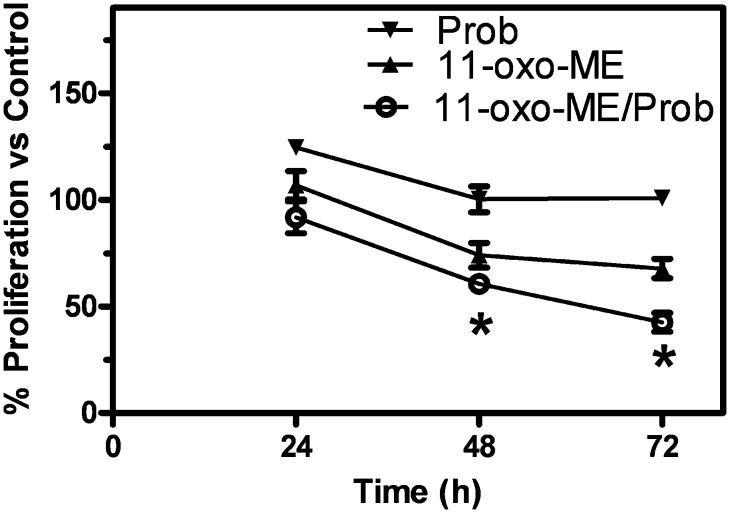
Cell proliferation of LoVo measured by MTT assay over multiple days with 2 μM 11-oxo-ETE-ME and/or 1 mM probenecid in 0.25% DMSO compared with vehicle control. Data are plotted as the means (n = 4) with SEM, representative of at least two independent experiments. Statistical significance was assessed by one-way ANOVA with post-hoc Bonferroni multiple comparison test (*P < 0.05).

**Fig. 8.**
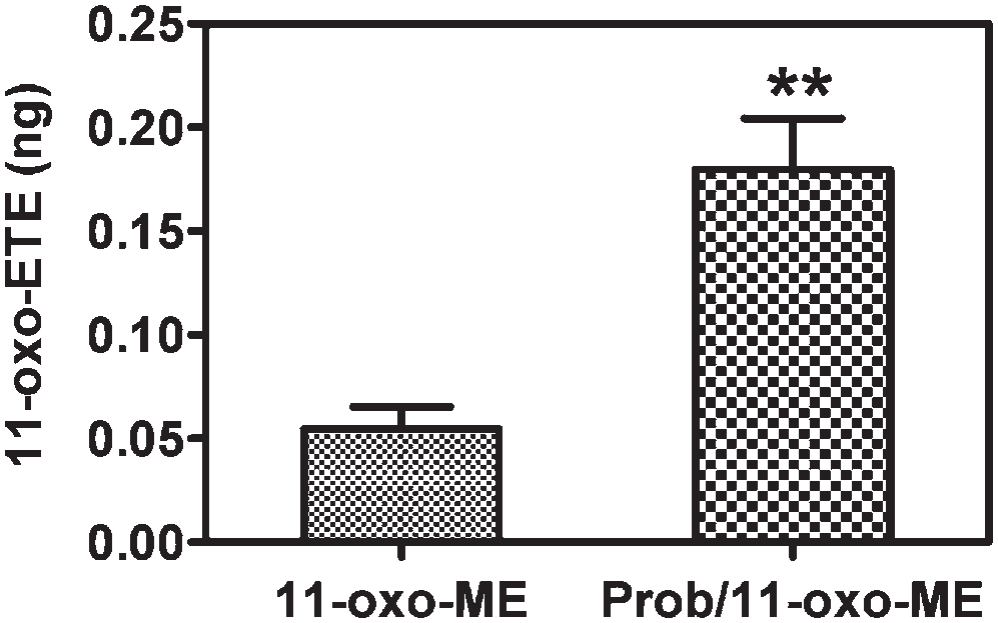
SID-LC-ECAPCI/SRM/MS quantification of the recovery of 11-oxo-ETE in LoVo cells. Cells were incubated with either 0.25% DMSO or 1 mM probenecid for 30 min before incubation with 11-oxo-ME (10 µM) in 0.25% DMSO vehicle control for 60 min. Cellular and media fractions were collected, extracted, spiked with [^13^C_20_]15-oxo-ETE, and then derivatized with PFB. Data are plotted as the means of triplicates with SEM. Statistical significance was assessed by two-way Student *t*-test (**P < 0.01).

To test the possibility of blocking the drug transporters to increase antiproliferative effects, a MTT assay over multiple days using the LoVo cell line was carried out. Treatments with probenecid, 11-oxo-ETE-ME, or the combination of both were compared with vehicle control arbitrarily set at 100%. At both 48 and 72 h, significantly increased antiproliferative effects were observed for the combination treatment versus either treatment alone (**Fig. 7**). Pretreatment with probenecid increased the recovery of 11-oxo-ETE from the 11-oxo-ETE-ME-treated LoVo cells (**Fig. 8**). This finding suggests that increased intracellular 11-oxo-ETE was the mechanism for the synergistic action of probenecid on 11-oxo-ETE-ME antiproliferative action.

## DISCUSSION

The involvement of COX-2 and 15-PGDH in cancer progression has been well documented (2, 28–33). Proproliferative AA metabolites derived from COX-2, such as PGE_2_ acting via the G-protein coupled PGE receptors (EP)_1_, EP_2_, and EP_4_, induce proliferation and angiogenesis (34–36). Autocrine and paracrine signaling of PGE_2_ in cancer leads to a feed-forward loop modulating local immune responses and increasing angiogenesis and proliferation (28, 31). A decrease in catabolic 15-PGDH leads to increased activity of PGE_2_ due to its reduced metabolic clearance (2). However, AA metabolism leads to a plethora of metabolites with distinct and sometimes opposing functions (37). Considerable work on the antiproliferative effects of AA metabolites have focused primarily on 15d-PGJ_2_ (38). These studies have been complicated by contradictory results (23, 39, 40), uncertainty over the actual physiological relevance of the tested compounds (41), and lack of dysregulation in disease states (42). Other cyclopentenone PGs, such as PGA_2_, have been linked to antiproliferative action via inhibition of the cell cycle through cyclin D1 at 100 μM (43).

11-oxo-ETE and 15-oxo-ETE are known endogenous compounds isolated from clinical specimens and are major metabolites of AA via COX/15-PGDH (10, 13, 14, 17, 18). In this study, measurable antiproliferative effects were seen in three of four tested cell lines at 2 μM and in all four cell lines at 10 μM (Fig. 4). Although 11-oxo-ETE was clearly more potent than 15-oxo-ETE, the effect of both eicosanoids was significant, and their effects could be modulated by targeted intracellular delivery or pharmacological blockade of transporters (Figs. 5–7). The amounts of 11-oxo-ETE and 15-oxo-ETE that were detected in the LoVo cells and HUVECs represented only a small fraction of the total amount of each oxo-ETE or oxo-ETE-ME that was added to the cells. From our previous work, we suspect that major amounts of the oxo-ETEs are conjugated to glutathione, exported, and cleaved to the cysteinyl-glycine adduct (10). We are actively investigating other biotransformation pathways that contribute to metabolic clearance. The finding that intracellular delivery of 11-oxo-ETE through use of the methyl-ester derivative increased the antiproliferative effects in LoVo cells (Figs. 4, 5, 7) lends support to the hypothesis that a plausible mechanism of action may be through intracellular targets. This was particularly evident in the increased antiproliferative activity of 11-oxo-ETE-ME (Fig. 5B) compared with the free 11-oxo-ETE (Fig. 5A). The amplification of antiproliferative effects and increased recovery of 11-oxo-ETE with probenecid cotreatment (Fig. 7) also supports this hypothesis. This is in agreement with an expanding body of work supporting a hypothesis for the mechanism of action for certain bioactive lipids through intracellular-signaling mediators (19, 22–24). These findings, along with our previous work on the GSH-mediated metabolism of 11-oxo- and 15-oxo-ETE may implicate intracellular uptake as a rate-limiting factor in bioactivity and metabolism of these compounds (10).

During tumorigenesis, significant upregulation of COX-2 occurs, which would increase the production of proproliferative PGE_2_ (31, 44) as well as the antiproliferative oxo-ETEs. However, there is also significant downregualtion of 15-PGDH (2, 5, 45–47), which would result in increased activity of PGE_2_ due to its decreased catabolism, coupled with a decrease in the formation of the oxo-ETEs (Fig. 1B) (13). Increased expression of MRP4 (27, 48, 49), the transporter involved in the efflux of PGE_2_ (50, 51), also occurs during tumorigenesis. This would further prevent the 15-PGDH-mediated metabolism of PGE_2_ in epithelial cells and further facilitate an increase in its activity at relevant membrane EPs. In contrast, increased efflux of oxo-ETEs mediated by MRP4 would result in reduced activity because (as described above) they have intracelluar targets. Finally, the upregulation of glutathione biosynthesis and increased glutathione-*S*-transferase expression (52–54) would result in increased conversion of oxo-ETEs into their corresponding inactive glutathione adducts (10). Therefore, tumor progression is associated with substantial activation of proproliferative PGE_2_ and metabolic inactivation of the oxo-ETEs.

## Supplementary Material

Supplemental Data
