# Supplementary material for: Cellular uptake and antiproliferative effects of 11-oxo-eicosatetraenoic acid
Source: J Lipid Res. 2013 Nov;54(11):3070–7. doi: 10.1194/jlr.M040741 (PMC3793611; doi:10.1194/jlr.M040741)
Supplement: Supplemental Data [file supp_54_11_3070__index.html]

Cellular uptake and anti-proliferative effects of 11-oxo-eicosatetraenoic acid — Cellular uptake and antiproliferative effects of 11-oxo-eicosatetraenoic acid — Supplemental Data 

# Cellular uptake and antiproliferative effects of 11-oxo-eicosatetraenoic acid

## Supplemental Data

**Files in this Data Supplement:**

- Supplementary Figure 1 - Treatment of LoVo cells with [13C20]-15-oxo-ETE. LoVo cells were cultured as described in the Methods section and then treated as described with 10 mM [13C20]-15-oxo-ETE. Cell and media fractions were pooled, and derivatization and analysis were performed as described in the Methods section except the internal standard was omitted. No detectable 11-oxo-ETE of 15-oxo-ETE was found, indicating that the [13C20]-15-oxo-ETE did not induce generation of 11-oxo-ETE or 15-oxo-ETE.
- Supplementary Figure 2 - Internal standard blank. 10 ng of PFB-derivatized [13C20]-15-oxo-ETE internal standard analyzed by normal phase LC-ECAPCI/MS. Noise levels in the 11-oxo-ETE and 15-oxo-ETE channels are shown to establish a baseline.
- Supplementary Figure 3 - Analysis of LoVo cell wash. The fourth wash of the cellular fraction was pooled from 11-oxo-ETE, 15-oxo-ETE, 11-oxo-ETE-ME and 15-oxo-ETE-ME treatments of LoVo cells, derivatized, and analyzed as described in the Methods section. 11-oxo-ETE and 15-oxo-ETE channels had levels of signal similar to those observed in an internal standard blank, indicating that the wash steps were sufficient to remove detectable 11-oxo-ETE and 15-oxo-ETE.
