# Supplementary figures and images for: Cellular uptake and antiproliferative effects of 11-oxo-eicosatetraenoic acid
Source: J Lipid Res. 2013 Nov;54(11):3070–7. doi: 10.1194/jlr.M040741 (PMC3793611; doi:10.1194/jlr.M040741)

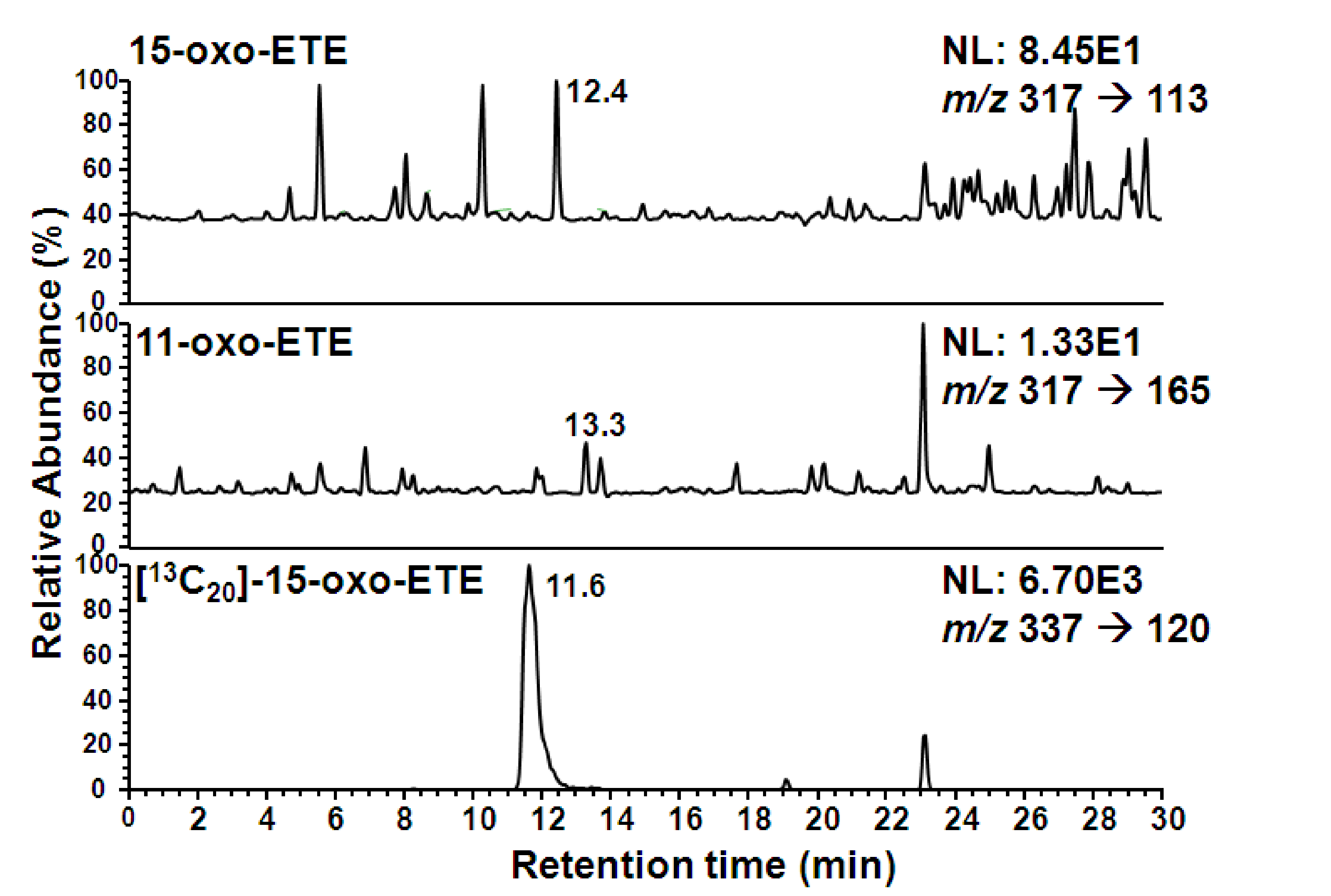

Supplement: Supplemental Data [file supp_M040741_jlr.M040741-1.doc]

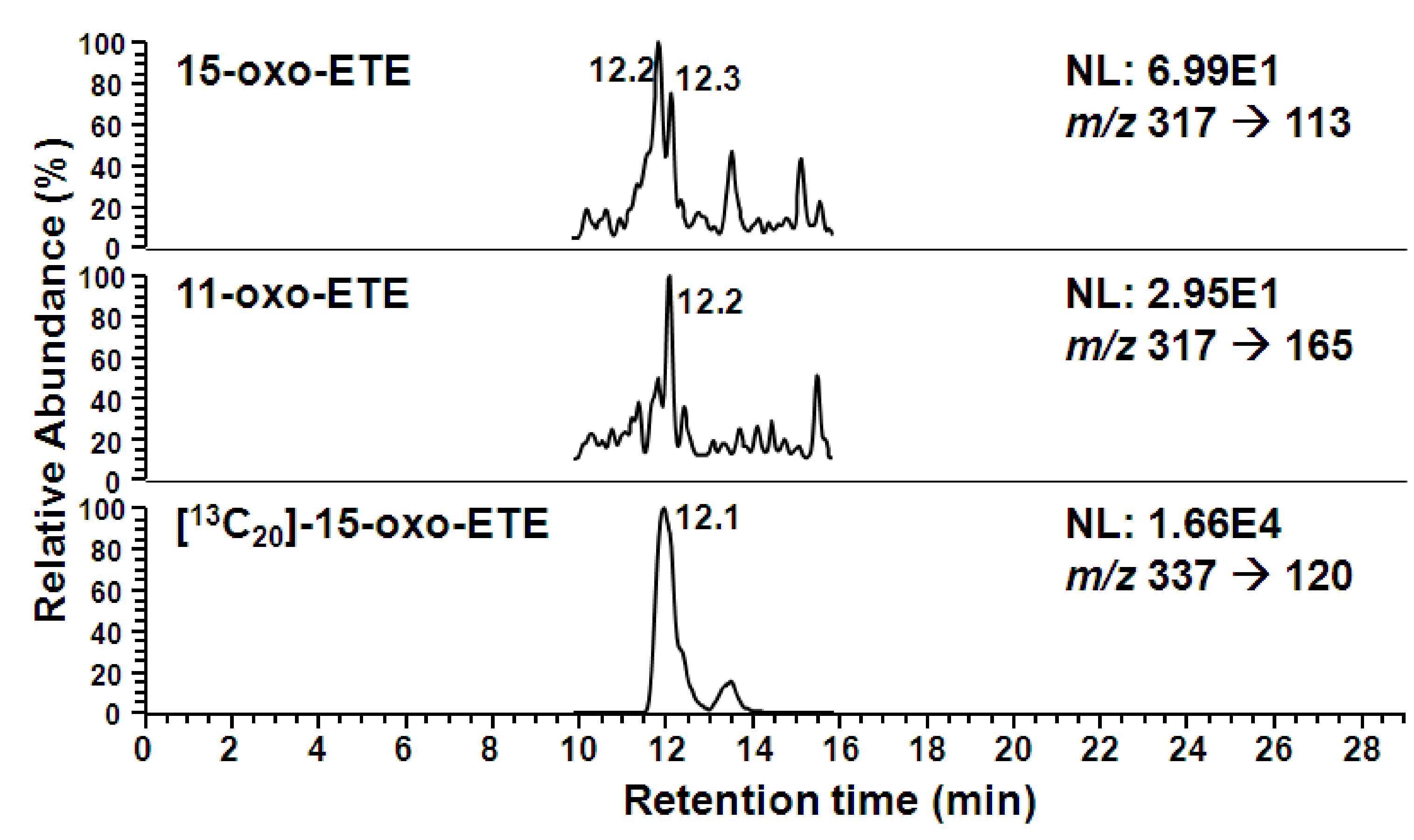

Supplement: Supplemental Data [file supp_M040741_jlr.M040741-2.doc]

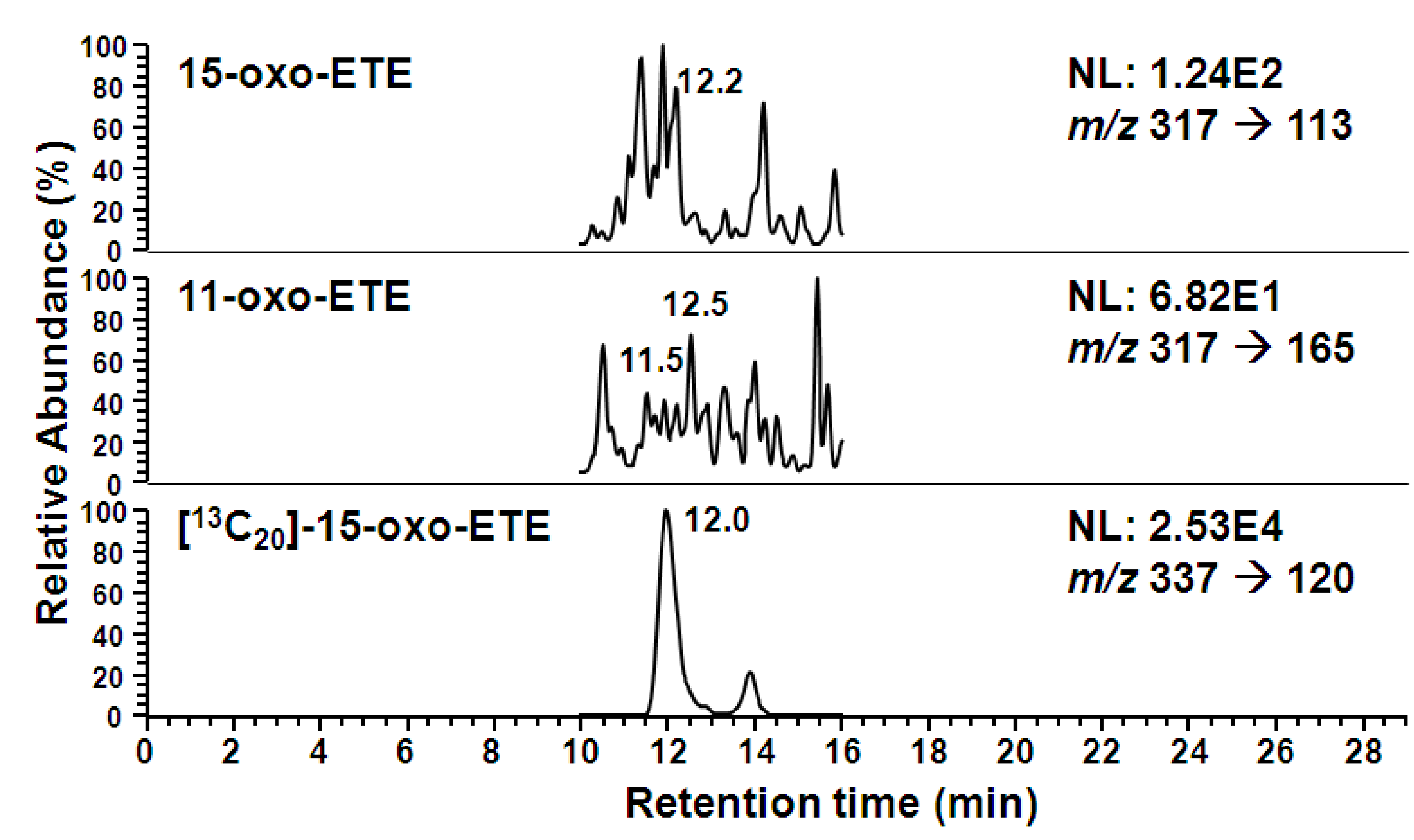

Supplement: Supplemental Data [file supp_M040741_jlr.M040741-3.doc]
